# Case report: Durable response to alectinib in *ALK*-rearranged lung adenocarcinoma with acquired, crizotinib-resistant *ALK* C1156F mutation

**DOI:** 10.3389/fonc.2022.915502

**Published:** 2022-09-20

**Authors:** Chuangzhou Rao, Liangqin Nie, Xiaokang Wu, Xiaobo Miao, Ting Chen, Liuxi Chen, Dongqing Zhang, Quan Lin

**Affiliations:** ^1^ Department of Radiotherapy and Chemotherapy, Hwamei Hospital, University of Chinese Academy of Sciences, Ningbo, China; ^2^ Department of Respiratory and Critical Care Medicine, The First Affiliated Hospital of Wenzhou Medical University, Wenzhou, China

**Keywords:** non-small cell lung cancer, ALK C1156, crizotinib, alectinib, acquired resistance

## Abstract

Treatment of *ALK*-rearranged non-small cell lung cancer (NSCLC) with tyrosine kinase inhibitors (TKIs) is challenged by the almost inevitable emergence of therapeutic resistance. Different profiles of resistance mechanisms have been reported for the currently available ALK TKIs. The *ALK* C1156Y mutation is reported in 2% of patients with acquired resistance to crizotinib. A rare substitution at the same site, C1156F, remains largely unknown. Existing evidence includes identification of C1156F and G1202R in an alectinib-resistant patient and sensitivity to crizotinib and resistance to later-generation 3ALK inhibitors in preclinical models. In this report, we present two cases in which NSCLC patients acquired the *ALK* C1156F mutation on crizotinib monotherapy. Both patients were men, and one had been heavily treated with chemotherapeutic regimens before identification of *ALK* rearrangement, whereas the other received crizotinib as first-line treatment. Genomic profiling of blood biopsies after progression on crizotinib suggested emergence of the *ALK* C1156F variant. Both patients subsequently received and responded favorably to alectinib, achieving respective progression-free survival of 21 and 15 months as of the latest follow-ups. To the best of our knowledge, this work is the first to provide clinical evidence of resistance to crizotinib and sensitivity to alectinib in NSCLC patients harboring acquired *ALK* C1156F mutation.

## Introduction

Tyrosine kinase inhibitors (TKIs) inhibiting the kinase activity of anaplastic lymphoma kinase (ALK) have become the standard-of-care for advanced non-small cell lung cancer (NSCLC) patients harboring oncogenic *ALK* rearrangement. In addition to the first-generation ALK TKI crizotinib, second-generation alectinib, ceritinib and brigatinib as well as third-generation lorlatinib have been approved by major regulatory bodies for *ALK*-positive NSCLC in the first- and/or later-line setting. However, therapeutic resistance almost invariably emerges and leads to disease progression (1).

Known mechanisms include *ALK* mutation, *ALK* amplification, and activation of bypass signaling. Secondary point mutations in the ALK kinase domain constitute the most common cause, appearing in approximately 20% of patients after progression on first-line ALK TKI and up to 70% for those under sequential TKI treatment (2). A diverse range of acquired drug-resistant mutations have been discovered, and the portfolios vary for different TKIs (2, 3). After progression, therapeutic options include definitive local therapy for limited lesions, continuing a second- or third-generation inhibitor, and chemotherapy (4). Elucidating the sensitivity profiles of individual mutations therefore facilitates choice of targeted therapy. *ALK* C1156Y occurred in 2% patients who had progressed on crizotinib (2) but not in those showing resistance to alectinib, brigatinib or lorlatinib (2, 5). Less is known regarding a rarer substitution on the same site, C1156F. This mutation was reported along with *ALK* G1202R in an *ALK*-positive NSCLC patient who progressed on crizotinib and alectinib (6). Since G1202R is a known mechanism of resistance found in 29% patients progressing on alectinib, this finding was inconclusive as to the sensitivity of the C1156F mutant to alectinib (2). Relevant preclinical data proposed the C1156F mutant as poorly druggable or undruggable by all available ALK TKIs except for crizotinib (7), and another study further supported resistance to lorlatinib in Ba/F3 cells (3). Together, existing evidence appears to favor sensitivity to crizotinib and resistance to later-generation ALK TKIs for the C1156F mutant.

Herein, we report two cases in which genetic testing with biopsies before and after disease progression on crizotinib identified *ALK* C1156F as an acquired resistant mutation. Both patients subsequently received and responded favorably to alectinib, achieving respective progression-free survival (PFS) of at 21 and 15 months. To the best of our knowledge, this report is the first to provide clinical evidence of resistance to crizotinib and sensitivity to alectinib in NSCLC patients harboring the ALK C1156F mutation.

## Case presentation

### Patient 1

**Figure 1:**
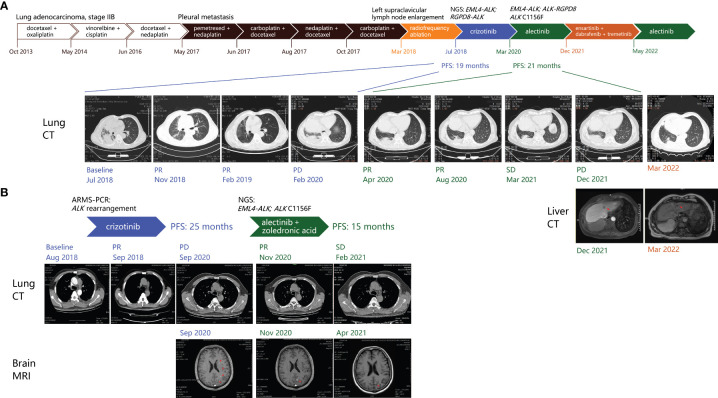
Course of management for **(A)** patients 1 and **(B)** 2 and representative imaging findings by brain MRI and pulmonary and liver CT scans since the start of ALK-targeted therapy. Red arrowheads indicate lesions. CT, computed chromatography. MRI, magnetic resonance imaging. PD, progressive disease. PR, partial response. Red arrowheads indicate brain lesions detected on MRI.

**Figure 2:**
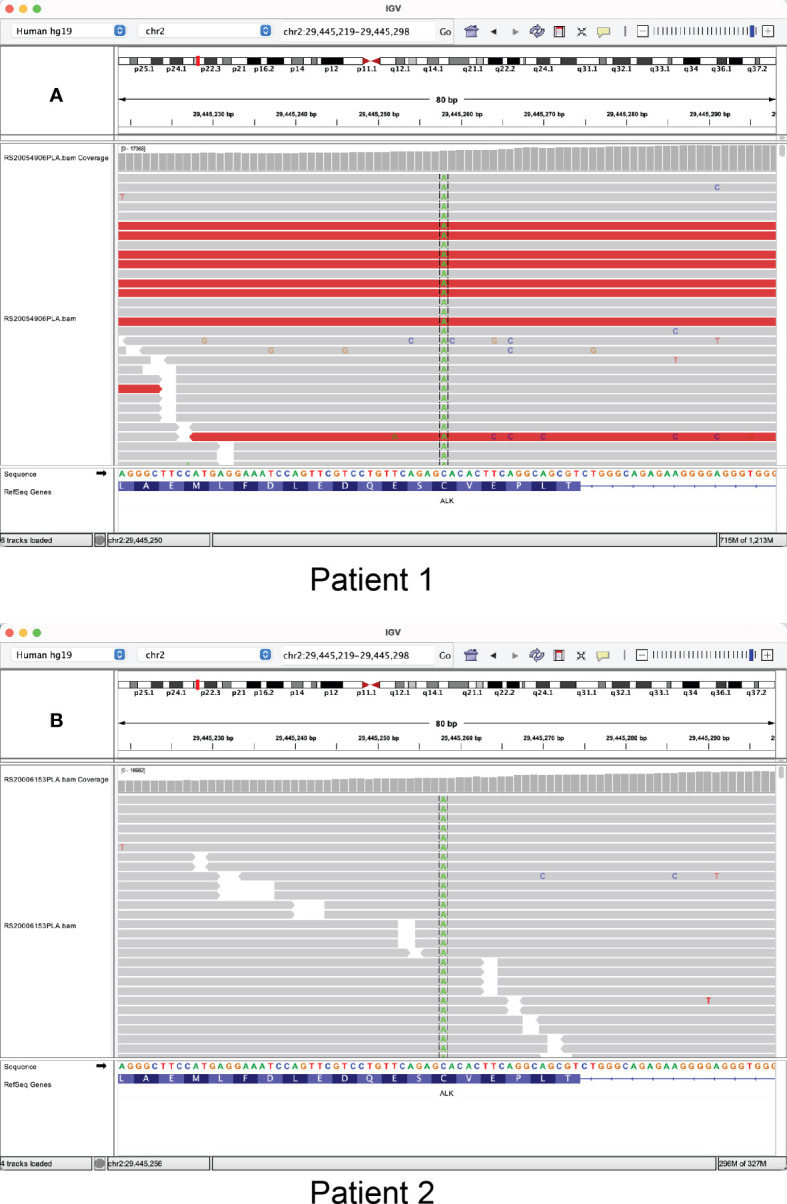
Integrative Genomics View snapshots showing the *ALK* C1156F missense mutation identified by next-generation sequencing from **(A)** patient 1 and **(B)** patient 2.

A 65-year-old male smoker received thoracoscopic right middle lobectomy and mediastinal lymph node dissection after detection of a pulmonary nodule by computed chromatography (CT; **Figure 1A**) in 2013. Pathological review of the surgical specimen and pleural nodule biopsy indicated a stage IV lung adenocarcinoma with nodal metastasis and pleural invasion. The patient was then placed on a series of chemotherapy regimens and ultrasonography-guided percutaneous radiofrequency ablation (**Figure 1A**). In Jul 2018, he showed multiple bilateral pulmonary nodules, enlarged lymph nodes in the mediastinum and right hilum, and a left mediastinal space-occupying lesion measuring 4.1 cm in greatest dimension). The pleural effusion (PE) was biopsied and subjected along with plasma to genomic profiling with a targeted panel of 168 cancer-related genes (Burning Rock Biotech, Guangdong, China), which identified from both samples an actionable *EML4-ALK* (E14:A20) rearrangement and a novel *RGPD8-ALK* (Rintergenic:A20) fusion. The patient was then placed on crizotinib and showed reduction in the left lung nodules, mediastinal and hilar lymph nodes, and left mediastinal space-occupying lesion (3.3 cm), consistent with partial response (PR) per the RECIST 1.1 guidelines (**Figure 1A**). In Feb 2020, he showed signs of progressive disease manifested as emergence of left pulmonary nodules and growth in the mediastinal and hilar lymph nodes and the left mediastinal space-occupying lesion (3.9 cm; **Figure 1A**). Sequencing analysis with plasma detected *EML4-ALK* (E14:A20) fusion, *ALK-RGPD8* (A19:R5’UTR), and *ALK* C1156F missense mutation (variant allele frequency [VAF] 0.83%; **Figure 2A**). The patient was then placed on alectinib (600 mg BID) on Mar 2020 and manifested PR within two months, when CT scans showed marked reduction in the left pulmonary ground-glass opacity, PE, and the left mediastinal space-occupying lesion (**Figure 1A**). Disease control lasted until Dec 2021, when a low-density mass was found in the liver. Pleural and peritoneal metastases were also suspected. Molecular testing of the liver mass revealed a *BRAF* V600E mutation, and the patient was started on combination therapy with ALK TKI ensartinib (8), dabrafenib and trametinib. The regimen was not well-tolerated despite tumor response and dosage decrease. The patient was placed back on alectinib after progression since May 2022. Although he has not visited afterwards, his family reported good general condition as of July.

### Patient 2

A 53-year-old male never-smoker visited the hospital with complaints of left chest pain that had persisted for a week. He was soon after admitted due to shortness of breath and aggravated chest pain. CT scans showed multiple nodules in the left lower pulmonary lobe, left PE, and multiple hyperdense lymph nodes in the mediastinum and bilateral hila (**Figure 1B**). Pathologic review of the pleural drainage suggested poorly differentiated lung adenocarcinoma, which was subsequently found positive for *ALK* rearrangement by amplification-refractory mutation system - polymerase chain reaction (ARMS-PCR). Crizotinib was accordingly started and achieved a PFS of 25 months, when imaging studies showed an enlarged mediastinal lymph node (1.9 cm) and multiple enhancing foci in the brain and the T12 and L4 vertebrae (**Figure 1B**). Genomic profiling of the blood revealed *EML4-ALK* fusion (E13:A20) and *ALK* C1156F (VAF 2.04%; **Figure 2B**), and the patient was placed on alectinib (600 mg BID) for second-line treatment in Sep 2020. Zoledronic acid was also used to manage metastatic bone disease. The patient showed intracranial response within two months, manifested as shrinkage in the nodules in the left parietal lobe and cerebellum, while there was shrinkage in a major mediastinal lymph node (1.4 cm), consistent with a radiographic response of PR (**Figure 1B**). Follow-up brain MRI in December showed intracranial progression manifested as newly emerged enhanced foci in the parietal, frontal, and left occipital lobe and cerebellum. The PFS on alectinib was therefore 15 months. The patient received gamma ray irradiation for brain lesions and continued on alectinib. No sign of disease progression was noted as of the latest follow-up in Jul 2022.

## Discussion

Alectinib is currently the preferred frontline therapy for *ALK*-positive NSCLC due to potent ALK kinase activity inhibition, high CNS penetration, and favorable toxicity profile. First-line crizotinib is still recommended, for which case later-line systemic options include either continuing crizotinib or starting second-generation ALK TKIs (1). The choice of TKI is contingent on the identification of the mechanism of resistance. For the Cys1156 residue, the most common mutation C1156Y was found in 2% of patients who have progressed on crizotinib (2) but was absent in an analysis of alectinib-resistant patients. A less frequent mutation C1156S was also shown to confer resistance to crizotinib *in vitro* (9). The C1156F mutant showed a 39-fold increase in half-maximal inhibitory concentration (IC50) for alectinib compared with the wild-type counterpart in mutant Ba/F3 cells, and therefore was proposed as undruggable for alectinib (7). However, we herein present clinical evidence that contrasts with these preclinical findings, suggesting caution in interpreting preclinical findings regarding drug sensitivity.

We also provide evidence that suggests a secondary nature of the C1156F mutation. In patient 1, next-generation sequencing analysis with targeted panels covering the entire *ALK* genomic sequence was performed both at baseline using plasma and PE and after progression on crizotinib using plasma, and *ALK* C1156F detected only at the latter timepoint with VAF of 0.83%. For patient 2, PE was tested before receiving crizotinib with ARMS-PCR designed for detecting *ALK* rearrangements at predetermined breakpoints near exon 20, which was indicated by failure of PCR with primers designed against nearby *ALK* sequence (10). Since the primers did not probe sequences near the C1156 codon (exon 22), it is undetermined whether at baseline the patient harbored *ALK* C1156F, which was identified from a blood sample by next-generation sequencing after onset of crizotinib resistance. However, since *de novo* ALK kinase domain mutations are highly uncommon (<3% of cases) in ALK TKI-naïve *ALK*-positive lung adenocarcinomas, it is more likely that in both cases the C1156F mutation was acquired and possibly mediated resistance to crizotinib. Furthermore, detection of *ALK* C1156F was previously reported in an *ALK*-rearranged NSCLC patient after sequential progression on crizotinib and alectinib monotherapies and together with *ALK* G1202R (6). G1202R was more frequently detected after onset of resistance to alectinib (29% patients) than to crizotinib (2% patients) (2), so it is likely that C1156F, like in our case, emerged first and induced crizotinib resistance.

Similar to alectinib, it also remains to be clinically validated whether the *ALK* C1156F mutation confers resistance lorlatinib. Currently the only relevant evidence comes from Ba/F3 cells, which showed an IC50 for the C1156F mutant comparable with that of G1269A, a mutant identified in patients after progression on lorlatinib (3). Another piece of clinical evidence regarding the *ALK* C1156 substitutions in NSCLC indicated that a secondary L1198F mutation paradoxically negated the effect of C1156Y and resensitized the tumor to crizotinib (5). Whether the same applies to C1156F would make an interesting find.

## Conclusion

In summary, we present two cases in which *ALK*-rearranged metastatic NSCLC patients acquired ALK kinase domain mutation C1156F that induced therapeutic resistance to crizotinib. Both patients were placed on alectinib and manifested favorable response within two months. As of the latest follow-ups, the patients have achieved respective progression-free survival of 21 and 15 months on alectinib. To the best of our knowledge, this work is the first to provide clinical evidence that *ALK* C1156F mutation is as an acquired resistance mechanism to crizotinib in *ALK*-positive NSCLC patients and remains sensitive to alectinib.

## Data availability statement

The original contributions presented in the study are included in the article/supplementary material. Further inquiries can be directed to the corresponding author.

## Ethics statement

Written informed consent was obtained from the individual(s) for the publication of any potentially identifiable images or data included in this article.

## Author contributions

CR and QL conceived of and designed the study and collected the data. CR, LN, XW, and XM analyzed and interpreted the data. CR and QL wrote the manuscript. TC, LC, and DZ provided valuable intellectual input to the manuscript. All authors approved the final version of the manuscript and are accountable for all aspects of the work.

## Funding

This study was supported by Zhejiang Natural Science Foundation (Grant No. LY19H160023) and Ningbo Medical Key Discipline Oncology, China (Grant No. 2016019).

## Acknowledgments

We would like to thank the patients and their families for their support. We are also grateful to the Xiao Zou, Wenjie Sun, Jianxing Xiang, Jiaqi Chu, Danlu Jiang, Xinze Lv and Haiyan Li at Burning Rock Biotech for technical assistance.

## Conflict of interest

The authors declare that the research was conducted in the absence of any commercial or financial relationships that could be construed as a potential conflict of interest.

## Publisher’s note

All claims expressed in this article are solely those of the authors and do not necessarily represent those of their affiliated organizations, or those of the publisher, the editors and the reviewers. Any product that may be evaluated in this article, or claim that may be made by its manufacturer, is not guaranteed or endorsed by the publisher.
